# Protective effect of the DNA vaccine encoding the major house dust mite allergens on allergic inflammation in the murine model of house dust mite allergy

**DOI:** 10.1186/1476-7961-4-4

**Published:** 2006-02-20

**Authors:** Nacksung Kim, Soon Seog Kwon, Jaechun Lee, Sohyung Kim, Tai June Yoo

**Affiliations:** 1 Medical Research Center for Gene Regulation, Chonnam National University Medical School, Gwangju, Korea; 2 Department of Internal Medicine, Catholic University Medical School, Seoul, Korea; 3 Department of Internal Medicine, College of Medicine, Cheju National University, Jeju, Korea; 4 Division of Allergy/Immunology, Department of Medicine, University of Tennessee, Memphis, TN, USA

## Abstract

**Background:**

Vaccination with naked DNA encoding antigen induces cellular and humoral immunity characterized by the activation of specific Th1 cells.

**Objective:**

To evaluate the effects of vaccination with mixed naked DNA plasmids encoding *Der p *1, *Der p *2, *Der p *3, *Der f *1, *Der f *2, and *Der f *3, the major house dust mite allergens on the allergic inflammation to the whole house dust mites (HDM) crude extract.

**Methods:**

Three hundred micrograms of these gene mixtures were injected into muscle of BALB/c mice. Control mice were injected with the pcDNA 3.1 blank vector. After 3 weeks, the mice were actively sensitized and inhaled with the whole house dust mite extract intranasally.

**Results:**

The vaccinated mice showed a significantly decreased synthesis of total and HDM-specific IgE compared with controls. Analysis of the cytokine profile of lymphocytes after challenge with HDM crude extract revealed that mRNA expression of interferon-γ was higher in the vaccinated mice than in the controls. Reduced infiltration of inflammatory cells and the prominent infiltration of CD8+ T cells were observed in histology of lung tissue from the vaccinated mice.

**Conclusion:**

Vaccination with DNA encoding the major house dust mite allergens provides a promising approach for treating allergic responses to whole house dust mite allergens.

## Background

It has been reported that IgE-mediated inflammation to mites is associated with diseases such as asthma, allergic rhinitis, and atopic dermatitis and that the relatively minor determinants of house dust mite (HDM) allergens show IgE binding with 40% or more of allergic sera [1-4]. The most frequently implicated allergens are derived from *Dermatophagoides pteronyssinus *(*Der p*) and *Dermatophagoides farinae *(*Der f*) [5]. Targeting specific T lymphocytes that induce or regulate the allergic inflammation is one of the therapeutic goals in allergic disorders. Specific immunotherapy with crude extracts has been used mainly in treating HDM-induced allergy. However, it has limited efficacy. Recently, vaccinations with naked DNA encoding antigen were reported to induce long-lasting cellular and humoral immune tolerance [6,7]. Injection of plasmid DNA encoding T cell epitopes could suppress allergic reaction [8,9]. However, the potential barrier to T cell receptor-based immunotherapy for allergy is the apparent complexity of the allergen-specific T cell response in terms of epitope usage in individuals [10]. A recent literature search showed that injection of plasmid DNA encoding *Der p *5 in rat not only inhibits *Der p *5-specific IgE antibody production but also an allergic response such as histamine release and airway hyperresponsiveness to *Der p *5 [11]. However, *Der p *5 is not one of the major HDM allergens; thus, it has limited clinical application [3]. In this study, we investigated immune responses by gene vaccination with plasmid DNA encoding major HDM allergens (*Der p *1,2, and 3, and *Der f *1,2, and 3) to challenges with whole HDM crude extract in sensitized mice.

## Methods

### Animals

Twenty female BALB/c mice 6–8 weeks old were purchased from Jackson Laboratory (Bar Harbor, ME) and bred in the animal facility of the University of Tennessee Health Science Center. This study was performed in accordance with the PHS Policy on Humane Care and Use of Laboratory Animals and the NIH Guide for the Care and Use of Laboratory Animal Welfare Act (7 U.S.C. et seq.). The animal use protocol was approved by the Institutional Animal Care and Use Committee (IACUC) of the University of Tennessee.

### Plasmid construction

Total mRNA was isolated from *Der p *and *Der f *HDM, respectively. By using murine leukemia virus reverse transcriptase and random hexanucleotide primer following the instructions of the Perkin Elmer Gene Amp RNA PCR kit (Perkin Elmer, Branchberg, NJ), first-strand cDNA was generated from 1 μg of total RNA and subjected to reverse transcriptase polymerase chain reaction (RT-PCR). The cDNA was used in PCR with Taq polymerase with primers specific for *Der p *1 (5'- CCGGAATTCGCCGCCACCATGGAAACTAACGCCTGCAGTATCAATGGA -3' and 5'- TGCTCTAGATTAGAGAATGACAACATATGGATATTC -3'), *Der p *2 (5'- CCGGAATTCGCCGCCACCATGGATCAAGTCGATGTCAAAGATTGTGCC -3' and 5'- TGCTCTAGATTAATCGCGGATTTTAGCATGAGTAGCAAT -3'), *Der p *3 (5'- CCGGAATTCGCCGCCACCATGATTGTTGGTGGTGAAAAAGCATTAGCTG -3' and 5'- TGCTCTAGATTACTGTGAACGTTTTGATTCAATCCAATCGATA -3'), *Der f *1 (5'- CCGGAATTCGCCGCCACCATGGAAACAAGCGCTTGCCGTATCAATTCG -3' and 5'- TGCTCTAGATTAGAGGTTGTTTCCGGCTTGGAAATATCCG -3'), *Der f *2 (5'- CCGGAATTCGCCGCCACCATGGATCAAAGTCGATGTTAAAGATTGTGCC -3' and 5'- TGCTCTAGATTAATCACGGATTTTACCATGGGTAGCAAT -3'), and *Der f *3 (5'- CCGGAATTCGCCGCCACCATGATTGTTGGTGGTGTGAAAGCACAAGCC -3' and 5'- TGCTCTAGATTACTGTGAACGTTTTGATTCAATCCAATCGAC -3'). These primers cover the mature excreted region of each gene and include EcoR1 and Xb1 sites for cloning. The amplified PCR products were subcloned into pcDNA3.1 eukaryotic expression vector (Invitrogen, San Diego, CA) and then sequenced to verify the insertion of the correct gene with the appropriate open reading frame.

### DNA preparation and vaccination

Each plasmid construct was prepared using Maxi prep (Quiagen, Chatsworth, CA). Mice were vaccinated by injection with 300 μg of pcDNA3.1 blank vector in 100 μl of phospate-buffered saline (PBS) (the control group) or the same amount of the mixed naked DNA encoding the major HDM allergens (the vaccination group) three times at weekly intervals into muscle (week 0, 1, and 2).

### Immunization and inhalation of allergen

Mice were sensitized with HDM crude extract previously described [12]. HDM crude extract was emulsified with an equal volume of complete Freund adjuvant (CFA) for immunization. Three weeks after the last vaccination, mice were sensitized subcutaneously at the base of the tail with 100 μg of HDM extract in CFA. The mice were also given an intraperitoneal dose of 300 ng of purified pertussis toxin at 24 and 72 h after first immunization. Seven days later, the mice were boosted again with the same amount of antigen in incomplete Freund adjuvant. Under inhaled anesthesia with methoxyflurane, mice were challenged with 10 μg of HDM crude extract through one nostril six times at weekly intervals after immunization.

### Determining total IgE, HDM-specific IgE, and HDM-specific IgG

The blood from the six mice in two groups was collected six times at week 0 (first vaccination), 3, 5 (first immunization), 7, 9, and 11. The HDM-specific IgG was determined by ELISA. One hundred microliter of HDM (5 μg/ml in 0.1 M carbonate buffer, pH 9.6) were dispensed in each well of a polystyrene microtiter plate (Cost, Cambridge, MA) and incubated overnight at 4°C. The concentration of HDM was determined by the preliminary experiments. The antigen-coated plates were washed three times in 0.05% PBS-Tween 20 buffer (washing buffer) and incubated with mice sera overnight at 4°C. The plates were washed five times with washing buffer and incubated with peroxidase-conjugated anti-mouse IgG antibody (Sigma, St. Louis, MO) overnight at 4°C. The plates were washed five times before adding citric acid-phosphate buffer (pH 5.0) containing 0.15 mg/ml of O-phenylenediamine (Sigma, St. Louis, MO). The color was developed at room temperature, and the reaction was stopped by 2.5 M sulfuric acid. The color was measured at 492 nm (Bio-Rad, Richmond, CA).

The total IgE level was determined by ELISA. One hundred microliter of anti-mouse IgE capture monoclonal antibody (mAb) (clone R35–72; Pharmingen, San Diego, CA) were added in each well to plates and incubated overnight at 4°C. After washing, 200 μL of 10% fetal calf serum were incubated at room temperature for 30 min. The plates were washed five times with washing buffer and incubated with the diluted mouse serum overnight at 4°C, followed by adding 100 μL of HRP-conjugated anti-mouse IgE detection mAb (clone R35–118; Pharmingen, San Diego, CA) overnight at 4°C. After washing, color was developed following the procedure for IgG. The purified mouse serum IgE (BD Biosciences, Palo Alto, CA) was used for total IgE standard. To measure HDM-specific IgE, the plate was coated with 25 μg/ml HDM in 0.1 M carbonate buffer (pH 9.6), and serum samples were diluted fivefold in 10% FCS. The concentration of HDM was determined by the preliminary experiments. The procedure after this point was the same as that for measuring HDM-specific IgG. The level of HDM-specific IgE was referenced to the standard serum pooled from six mice that were immunized with 100 μg of HDM twice and inhaled with 10 μg of antigen six times. The standard serum was calculated as 100 ELISA units/ml.

### Immunohistochemical staining for CD4+ and CD8+ T cells in lung tissue

The lung tissues from the vaccination and control groups were removed immediately after the final intranasal inhalation. Tissues were fixed with periodate-lysine-paraformaldehyde solution for 24 h at 4°C. The specimens were rinsed with 0.01 M of PBS (pH 7.4) containing 10% to 20% sucrose for 36 h at 4°C, embedded in OCT compound (Miles Laboratories Inc., Elkhart, IN), and immediately frozen. The lung specimens were immersed in 10% EDTA and decalcified for 10 days at 4°C. Frozen sections cut at 4 to 6 μm in thickness were dehydrated and rinsed in cold PBS. The endogenous pseudoperoxidase was blocked with absolute methanol containing 0.5% hydrogen peroxide for 20 min at room temperature. The sections were treated with 10% normal goat serum in PBS to reduce nonspecific binding. Biotin conjugated rat anti-mouse CD8 or CD4 mAb (Pharmingen, San Diego, CA) diluted to 1:200 in PBS containing 0.5% bovine serum albumin was applied to the sections and incubated overnight at 4°C. After rinsing, the sections were incubated with avidin-biotin peroxidase complexes (Vectastain Elite ABC Kit, Vector Laboratories Inc., Burlingame, CA) for 30 min at room temperature and rinsed sufficiently with PBS. The reaction was developed with 0.02% 3,3'-diaminobenzidine in 0.05 M of Tris buffer (pH 7.6) with 0.005% hydrogen peroxidase for 7 min. The sections were dehydrated, cleared in xylene, and mounted.

### Histological examination of lung tissue

Mice were anesthetized with a mixture of ketalar (35 mg/ml), rompun (0.6%/ml) and atropine (0.1 mg/ml), of which 0.2 ml was injected intramuscularly. The vascular bed of the lungs was perfused with 0.01 M PBS and then with 4% paraformaldehyde 0.1 M PBS buffers. Whole lungs were taken out and stored in 4% paraformaldehyde for 24 h at 4°C. After fixation, these tissues were dehydrated and embedded in paraffin. Frozen sections cut at 3 μm in thickness were stained by hematoxylin and eosin. After coding, the sections were evaluated by two observers using light microscopy. The amount of inflammation per section was scored using the method described by Hessel et al. [13]. Lungs that showed no focal inflammation were scored as grade 0. Those that showed one or two centrally located microscopic foci of inflammatory infiltrate were graded as 1. In grade 2, a dense inflammatory infiltrate was seen in a perivascular and peribronchial distribution originating in the center of the lung. In grade 3, the perivascular and peribronchial infiltrates extended to the periphery of the lung.

### Measuring cytokine mRNA expression

Measuring the expression level was done as previousy described [9]. Briefly, four mice from each group were sacrificed 10 days postboost. The lymph nodes were removed from the mice and minced to create single cell suspensions. Cells were cultured in RPMI for 18 h with no antigen as a negative control, recombinant *Der p *1 (100 μg/ml), or HDM crude extract (100 μg/ml). Cells were washed with PBS buffer and mRNAs prepared (Biotech, Houston, TX). By using murine leukemia virus reverse transcriptase and random hexanucleotide primer following the instructions of the Perkin Elmer Gene Amp RNA PCR kit (Perkin Elmer, Branchber, NJ), first-strand cDNA was generated from 1 μg of total RNA and subjected to RT-PCR analysis. We used the primers specific for β-actin (5'- GTGGGCCGCTCTAGGCACCAA -3' and 5'- CTCTTTGATGTCACGCACGATTTC -3') as control primer, IL-2 (5'- TTCAAGCTCCACTTCAAGCTCTACAGCGGAAG -3' and 5'- GACAGAAGGCTATCCATCTCCTCAGAAAGTCC -3'), IFN-γ (5'- TGCATCTTGGCTTTGCAGCTCTTCCTCATGGC -3' and 5'- TGGACCTGTGGGTTGTTGACCTCAAACT TGGC -3') (Clonetech, Palo Alto, CA), IL-4 (5'- CAGCTAGTTGTCATCCTGCTCTTC -3' and 5'- GTGATGTGGACTTGGACTCATTCATGG -3'), or IL-5 (5'- TGTCTGGGCCACTGCCATGGAGATTC -3' and 5'- CCATTGCCCACTCTGTACTCATCACAC -3') in the RT-PCR analysis. The amplified DNAs of β-actin, IFN-γ, IL-2, IL-4, and IL-5 were 540, 365, 413, 354, and 349 base pairs, respectively.

### Statistical analysis

Data in immunoglobulin response were analyzed by Student's paired *t *test for comparisons between control and vaccination groups. Histological grades were analyzed by a nonparametric Mann-Whitney U test. Data were expressed as mean ± SD. A *p-*value of < 0.05 was considered significant.

## Results

### Downregulation of allergen specific IgE production by DNA vaccination

**Figure 1:**
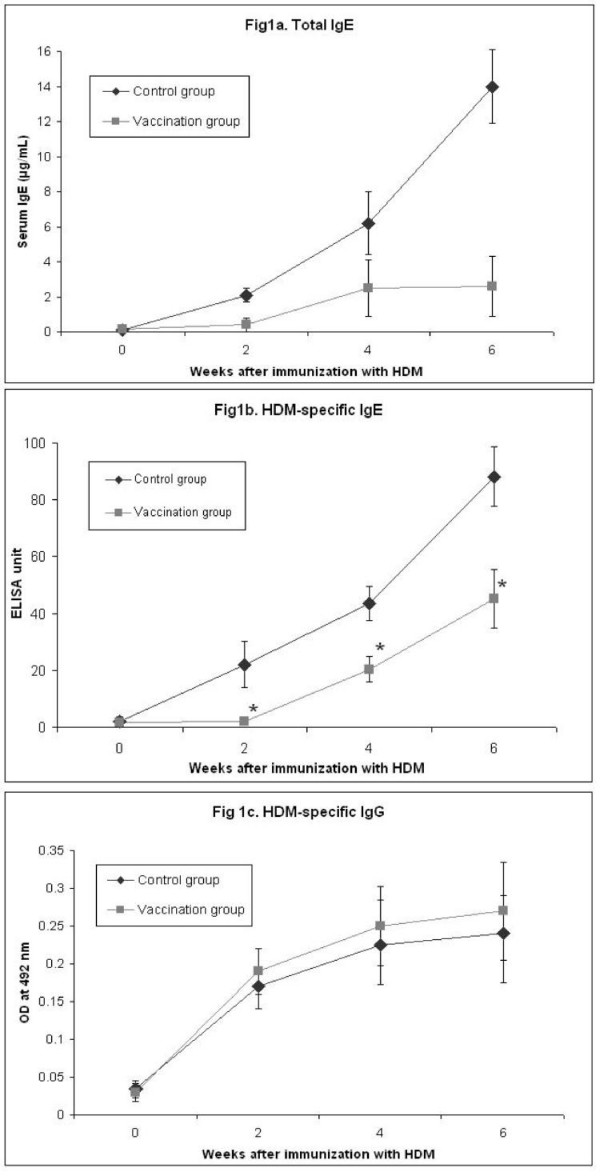
**Effect of vaccination on immunoglobulin production**. The total IgE antibody levels (1a), HDM-specific IgE antibody levels (1b), and HDM-specific IgG antibody levels (1c) in sera of each mouse were detected by ELISA every 2 weeks after immunization with HDM. The data are expressed as means ± SD (n = 6 per group). *P < .05 compared with the control group.

DNA vaccination with the major HDM allergen gene, *Der p *1, 2, and 3, and *Der f *1, 2, and 3 showed about 50% reduction of HDM-specific IgE and more than 70% reduction of total IgE compared with the control group at 6 weeks after immunization (Fig. 1a and 1b). However, production of HDM-specific IgG antibody showed no difference (Fig. 1c). Thus, *in vivo *total and allergen-specific IgE synthesis might be efficiently inhibited by DNA vaccination.

### Histological and immunohistochemical study

**Figure 2:**
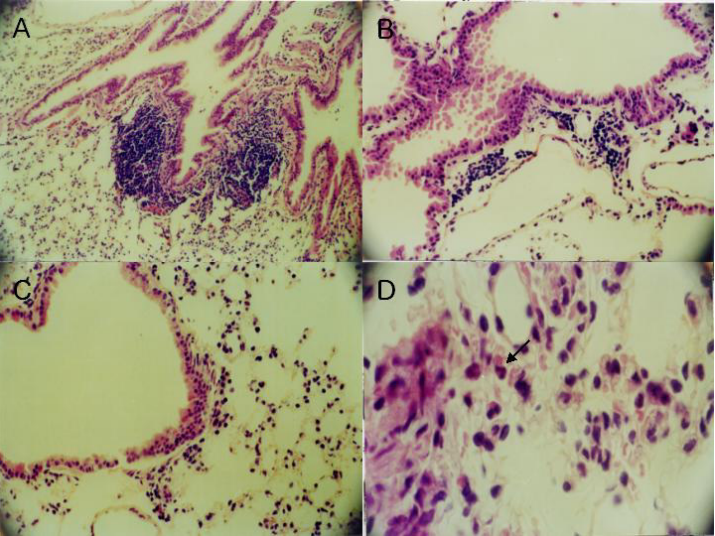
**Effect of genetic vaccination on lung histopathology in an animal model of allergy**. A and B, Light microscopic examinations of lung tissue from control group mouse (×100 and ×200). C, From vaccination group mouse (×200). D, Inflammatory cells including eosinophils (indicated with arrow) were observed in the peribronchial area in lung tissue from control group (×600). All tissue samples were stained with hematoxylin and eosin.

**Figure 3:**
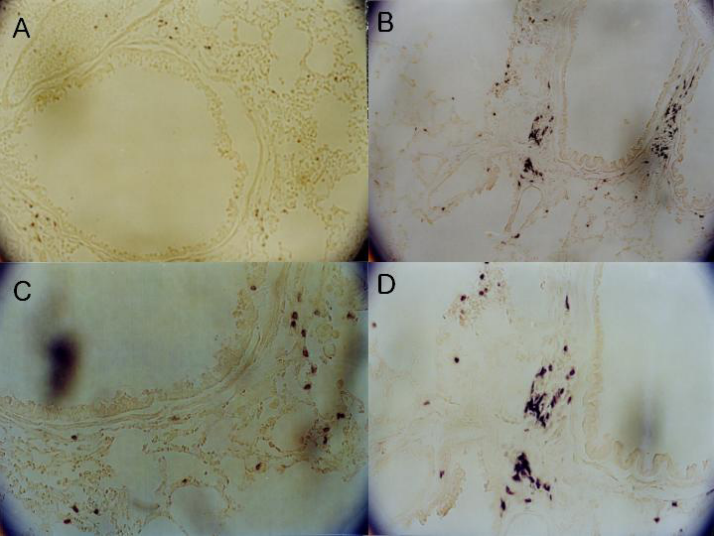
**Immunohistochemical examination for CD8+ T cells in lung tissue**. In the lung tissue from the vaccination group, more CD8+ T cells were infiltrated along the airway than in control group. A and B, Lung tissue from control group mouse (×100 and ×200). C and D, Vaccination group (×100 and ×200). Immunohistochemical staining with rat anti-mouse CD8 monoclonal antibody.

To investigate whether the DNA vaccination affects inflammation of lung, we stained lung tissue by histological and immunohistochemical methods. The lungs from the control group showed much more infiltration of inflammatory cells in the submucosa of airways than did those lungs from the vaccination group. The inflammation grades were scored as 1.64 ± 0.52 (mean ± SD) in the control group and 0.68 ± 0.48 in the vaccination group (Fig. 2a, 2b, and 2c). Also, eosinophils were detected in the lungs of the control mice (Fig. 2d). In the immunohistochemical stain for CD4 and CD8 molecules, the more CD8+ cells were infiltrated in the submucosa and mucosa of airway from the vaccination group compared with the control group (Fig. 3). But no difference in CD4+ cells was shown between the two groups. We considered whether the DNA vaccination might have an effect on the cellular response and the CD8+ T cells, which might protect against subsequent allergen challenges.

### Cytokines expressed by antigen stimulation

**Figure 4:**
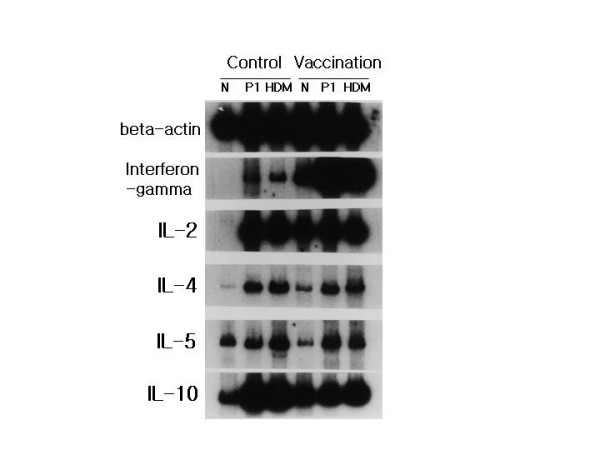
**Cytokine expression in lymphocytes**. Lymphocytes from the control group and vaccination group were cultured in the presence of no antigen (N), recombinant *Der p *1 (P1), and HDM crude extract (HDM) for 18 h. mRNAs of each indicated cytokine (interferon-gamma, IL-2, IL-4, IL5, IL-10) were measured by RT-PCR and that of beta-actin was measured for control.

Lymphocytes were harvested from lymph nodes of the two groups of mice and stimulated with recombinant *Der p *1 or HDM crude extract to determine the Th1 or Th2 cytokines involved in the DNA vaccination. Significantly elevated expression of IFN-γ mRNA was detected in the vaccination group compared with that in the control group. However, mRNA expression of IL-2, 4, 5, and 10 showed no difference from the control group (Fig. 4).

## Discussion

Diseases such as allergic asthma, rhinitis, and atopic dermatitis are all characterized by elevated levels of serum IgE. Total and specific IgE levels also show a close relationship with clinical symptoms in atopic allergy [14]. A variety of approaches targeting the suppression of IgE have been proposed such as synthetic peptides and T cell vaccine. However, synthetic peptides have substantial limitations because of poor immunogenecity [15,16]. Recently, in an animal model of allergic disorders, DNA vaccine encoding one of the major birch pollen allergens has been shown to be allergen-specifically protective and therapeutic [17]. DNA vaccination with plasmid encoding *Der p *5, one of the minor HDM allergens, was reported to prevent induction of specific IgE synthesis [11]. Vaccination with pDNA encoding *Der p *5 was shown to induce Th1 immune response to the encoded antigens. However, these results have some limitations on the clinical application for treating allergic disorders. Each allergen that causes allergic disorders in humans contains various kinds of protein that have their own epitopes and are complex. The *Der p *5 allergen reacts with about only 40% of allergic sera to HDM, and the *Der p *1 and 2 allergens react with about 80% of allergic sera [3,5]. To evaluate the effect of the gene vaccination with DNA fragments encoding major allergen on the allergic response to whole HDM extract, we used plasmid with cDNAs encoding the major six HDM allergens (*Der p *1, 2, and 3, and *Der f *1, 2, and 3) for vaccination. We showed about 50% reduction of HDM-specific IgE and more than 70% inhibition of total IgE at 6 weeks after immunization compared with control group. DNA vaccine with plasmid encoding the major HDM allergens might inhibit IgE synthesis more efficiently than encoding one of the HDM allergens.

In animal models of allergic disease, it has been established that Th2 responses are mediated by T helper cells that secret cytokines such as IL-4 and IL-5, which induce antibody production in B cells, and IgE plays a central role in allergic responses [18]. IFN-γ is the Th1 cytokine responsible for inhibiting IL-4-mediated IgE responses and promoting the formation of IgG2a [19]. Plasmid vector containing DNA that encodes allergens has been reported to decrease Th2-mediated responses, enhance Th1 responses, and suppress the allergic response [11,20,21]. In this investigation, mRNA expression of IFN-γ in lymphocytes from the vaccination group increased significantly relative to that from the control group, and less production of total and specific IgE in the vaccinated group was detected than in the control group, suggesting that the gene vaccination might successfully redirect the immune response from Th2 into Th1 to the encoded antigen or allergen.

Allergic asthma is characterized as a chronic inflammatory disease of the bronchi. It is well established that a variety of cells including mast cells, eosinophils, and lymphocytes play a role in this process [22,23]. After inhalation challenges, the inflammatory cells migrate from the peripheral blood to the site of inflammation in the bronchial mucosa, and Th2 type cytokines are dominantly detected in bronchoalveolar lavage fluid [24,25]. However, our investigation showed that DNA vaccination successfully reduced the recruitment of inflammatory cells in lung tissues. The effect of DNA vaccination in allergic inflammation might be elicited through not only humoral immune responses but also cellular responses.

T lymphocytes have been suggested to play a key role in orchestrating the interaction of the participating cells since they are able to release an array of cytokines that can attract, prime, and activate other cell types [25]. A successful outcome of immunotherapy is known to be associated with the development of regulatory T cells, which can downregulate the allergic response [26-30]. It is also known that functionally distinct subsets of CD8+ T cells may play an important regulatory role in IgE production [30-32]. However, there are some different explanations regarding the mechanisms of DNA vaccine. Manickan et al. [33] demonstrated the mechanism of genetic immunization against herpes simplex virus principally by CD4+ T cells, not by CD8+ T cells. Lee et al. [22] reported that both CD4+ and CD8+ subsets of T cells from pDNA vaccinated mice can suppress IgE antibody production by affecting the primary response or by propagating the Th1 memory response in a passive cell transfer system. Draghi et al. [34] investigated whether DNA vaccination leads to the generation of a distinct population of noncytotoxic/regulatory CD8+ T cells. In the authors' immunohistochemical investigation, more CD8+ T cells were more infiltrated in the lung tissue of the vaccination group than that of the control group.

Peptides derived from extracellular molecules are presented to CD4+ T cells by MHC (histocompatibility complex) class II molecules normally generated by antigen-presenting cells, whereas peptides derived from intracellular proteins are generally presented to CD8+ T cells by MHC class I molecules, which are expressed on virtually all somatic cells [35]. We injected mixed naked DNA into the muscle of the murine model of allergic disorder. Some of the injected DNA might be postulated to stay in the nuclei of cells or be integrated in the host DNA and elicit the endogenous production of an allergenic protein. MHC class I molecule, might induce CD8+ T cells that protectively function in immune response against a subsequent allergic challenge in sensitized host cells. The CD8+ T cells might be capable of conferring protection from allergic inflammation. DNA vaccination, which contains plasmid and DNA encoding specific allergen, might provide a more efficient therapeutic method for intervening allergic responses than conventional specific immunotherapy with allergen extracts.

## Abbreviations

HDM : house dust mite

pDNA : plasmid DNA

## Authors' contributions

N Kim and SS Kwon carried out animal and molecular experiments and initial draft. J Lee and S Kim handled images of the draft and final draft. TJ Yoo supported the whole step of experiment and submission.
